# Zincophorin – biosynthesis in Streptomyces griseus and antibiotic properties

**DOI:** 10.3205/id000026

**Published:** 2016-11-28

**Authors:** Elisabeth Walther, Sabrina Boldt, Hirokazu Kage, Tom Lauterbach, Karin Martin, Martin Roth, Christian Hertweck, Andreas Sauerbrei, Michaela Schmidtke, Markus Nett

**Affiliations:** 1 Jena University Hospital, Department of Virology and Antiviral Therapy, Jena, Germany; 2 Department of Biomolecular Chemistry, Leibniz Institute for Natural Product Research and Infection Biology, Hans Knöll Institute, Jena, Germany; 3 Technical University Dortmund, Department of Biochemical and Chemical Engineering, Dortmund, Germany; 4 Bio Pilot Plant, Leibniz Institute for Natural Product Research and Infection Biology, Hans Knöll Institute, Jena, Germany

**Keywords:** antibiotic, zincophorin, griseochelin, polyketide, Streptomyces griseus, Streptococcus pneumoniae

## Abstract

Zincophorin is a polyketide antibiotic that possesses potent activity against Gram-positive bacteria, including human pathogens. While a number of total syntheses of this highly functionalized natural product were reported since its initial discovery, the genetic basis for the biosynthesis of zincophorin has remained unclear. In this study, the co-linearity inherent to polyketide pathways was used to identify the zincophorin biosynthesis gene cluster in the genome of the natural producer *Streptomyces griseus* HKI 0741. Interestingly, the same locus is fully conserved in the streptomycin-producing actinomycete *S. griseus* IFO 13350, suggesting that the latter bacterium is also capable of zincophorin biosynthesis. Biological profiling of zincophorin revealed a dose-dependent inhibition of the Gram-positive bacterium *Streptococcus pneumoniae*. The antibacterial effect, however, is accompanied by cytotoxicity. Antibiotic and cytotoxic activities were completely abolished upon esterification of the carboxylic acid group in zincophorin.

## 1 Introduction

In times of increasing antibiotic resistance, natural products are rediscovered as an important source of potential drug leads [1]. Aside from *de novo* discovery programs [2], [3], the reevaluation of known natural products might be a promising approach to satisfy the urgent need for novel anti-infectives [4]. Some bacterial secondary metabolites, among them the lipopeptide daptomycin and the macrolide fidaxomicin, which were known since the 1980s, only lately made their way into the clinic [5], [6]. Thus, it is evident that even some ‘old’ compounds still hold the potential for becoming anti-infective drugs. 

**Figure 1:**
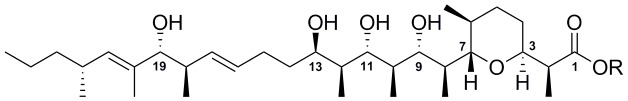
Structures of zincophorin (R=H) and its methyl ester (R=CH_3_).

In 1984, academic and industrial research groups independently reported the discovery of the polyketide zincophorin or griseochelin (Figure 1 (Fig. 1)) from the actinomycete *Streptomyces griseus* [7], [8]. This ionophore antibiotic inhibited the growth of several Gram-positive bacteria and was found to be particularly active against *Clostridium welchii*, which is nowadays referred to as *Clostridium perfringens*. Due to its intriguing structure and bioactivity, a number of synthetic routes to zincophorin and fragments of this natural product have been developed [9], [10]. Owing to the complexity of zincophorin’s structure, however, none of the described syntheses has been used for the generation of analogues to date. In order to clarify structure-activity relationships, an alternative and inexpensive approach for the structural modification of zincophorin would be desirable. Bioengineering strategies are particularly appealing for the diversification of polyketide natural products, including zincophorin, as they bypass the challenges of asymmetric total synthesis and may also lead to derivatives that are hardly accessible by semisynthesis [11], [12], [13]. However, they presume an identification of the involved biosynthetic genes.

The present communication discloses the gene cluster for the assembly of zincophorin, which was identified by genome sequencing and bioinformatic analysis of the native producer strain *S. griseus* HKI 0741. Furthermore, we provide the results from an extended testing of the antibiotic properties of zincophorin and its carboxylic acid methyl ester.

## 2 Materials and methods

### 2.1 Genome sequencing and assembly

For the isolation of high-molecular weight genomic DNA, the zincophorin-producing strain *S. griseus* HKI 0741 was cultured in R5 medium for three days at 30°C under aerobic condition. The mycelium was harvested from the actively growing culture by centrifugation and washed with Tris-EDTA (TE) buffer. Subsequently, the mycelium was stored at –20°C. The frozen cells were resuspended in TE buffer containing lysozyme (Sigma-Aldrich) and incubated at 37°C for 30 min. Afterwards, sodium dodecyl sulfate was added to a final concentration of 1%. The cell lysate was then extracted with phenol/chloroform. After centrifugation, genomic DNA was precipitated from the aqueous phase with isopropanol. The genomic DNA was dissolved in TE buffer containing RNase A (Novagen). After one hour of incubation at 37°C, proteinase K (Carl-Roth) was added and the incubation was continued for an additional hour at 37°C. Again, the sample was extracted with phenol/chloroform followed by isopropanol precipitation. The genomic DNA sample was dissolved in sterile water. Prior to whole genome sequencing the purity, quality and size of the isolated genomic DNA was assessed. The whole-genome sequence of *S. griseus* HKI 0741 was determined by two single-molecule real-time (SMRT) cell runs using PacBio RS II P4C2 technology and assembled by the hierarchical genome assembly process (HGAP) [14], [15], yielding a single linear contig of 8.1 Mbp.

### 2.2 Annotation and analysis of the zincophorin gene cluster

Contiguous sequences that harbor putative open reading frames for polyketide synthases were identified by sequence alignments with conserved domains of characterized biosynthesis genes following a previously described protocol [16]. Prediction of protein-coding open reading frames (ORFs) on these pre-selected contigs was carried out with FramePlot 2.3.2 [17]. Functional annotation was then performed by means of similarity searches against multiple protein databases by using a set of rules for assigning a specific product description depending on the search results [18]. Product descriptions were further manually refined. The annotated nucleotide sequence for the zincophorin gene cluster has been deposited in GenBank under accession number KT345957. 

### 2.3 General experimental procedures

IR spectra were recorded on a JASCO FT-IR (4100) spectrometer. High-resolution mass determination was carried out using a Finnigan TSQ quantum ultra mass spectrometer (Thermo Scientific). NMR spectra were recorded at 300 K on Bruker Avance III spectrometers with chloroform-*d_1_* as solvent and internal standard. The solvent signal was referenced to δ_H_ 7.24 ppm and δ_C_ 77.0 ppm, respectively. Analytical HPLC was performed on an Agilent 1100 Series LC/MSD trap. Flash column chromatography was undertaken using silica gel 60 M (230–400 mesh). TLC analyses were performed on silica gel plates (Sil G/UV254 0.20 mm, Macherey-Nagel) using a 9:1 mixture of chloroform and methanol as the eluent. Analytes were detected with vanillin-sulfuric acid spray reagent.

### 2.4 Fermentation and isolation of zincophorin

Lyophilized stock mycelia were used to inoculate 50 mL of the growth medium, consisting of 15 g L^–1^ soybean flour, 15 g L^–1^ glucose, 5 g L^–1^ NaCl, 1 g CaCO_3_, and 0.3 g L^–1^ KH_2_PO_4_. After incubation at 28°C on a rotary shaker for 24 h, aliquots (15 mL) of the pre-culture were transferred to 2-liter flasks containing 400 mL of the growth medium. The cultivation was continued for one day using the aforementioned conditions. Two liters of this culture were used to inoculate 50 L of the fermentation medium (40 g L^–1^ soybean flour, 50 g L^–1^ glucose, 2.5 g L^–1^ NaCl, 6 g L^–1^ CaCO_3_, 0.5 g L^–1^ KH_2_PO_4_, 6 g (NH_4_)_2_SO_4_, and 3 g L^–1^ FeCl_3_) in a 50-liter bioreactor. The fermentation was carried out for 4 days with aeration at 20 L min^–1^ and stirring at 200 rpm. The culture filtrate was separated from the mycelium by filtration. Subsequently, the mycelium was lyophilized and extracted with 2×10 L dichloromethane (DCM). The combined extracts were concentrated under reduced pressure. An initial fractionation of the oily residue was accomplished by column chromatography on silica gel 60 using a gradient of chloroform/methanol as eluent. Fractions containing zincophorin were further purified by column chromatography on silica gel 60 using *n-*hexane/ethyl acetate as the mobile phase and on Sephadex LH-20 using a 1:1 mixture of dichloromethane and methanol as eluant. The dried crude product was then dissolved in methanol and zincophorin was precipitated from this solution upon addition of destilled water. To release the free acid, 5 mg of the calcium salt were suspended in 5 N HCl (3 mL) and stirred for 3 h at room temperature. Zincophorin was extracted with DCM. After removal of residual water from the organic phase with anhydrous Na_2_SO_4_ and evaporation of the solvent, the free acid was obtained.

### 2.5 Preparation of zincophorin methyl ester

1.5 eq. of trimethylsilyldiazomethane (2 M in *n*-hexane, 0.5 mmol) was dissolved in 1 mL toluene/methanol (3:2) and added dropwise to an ice-cooled solution of 1 eq. zincophorin (20 mg, 0.4 mmol) in 3 mL toluene/methanol (3:2). The mixture was allowed to warm up and stirred for 2 h at room temperature. After evaporation of the solvent the oily residue was diluted in ethyl acetate and the organic phase was washed with saturated Na_2_CO_3_ and brine. The organic phase was dried over anhydrous Na_2_SO_4_ and the solvent was evaporated. The methyl ester of zincophorin was obtained as colorless oil. 

*Zincophorin methyl ester.* IR (film): 3385, 2956, 2929, 2870, 1734, 1456, 1380, 1276, 1120, 1079, 1016, 968 cm^–1^. ^1^H NMR (500 MHz, chloroform-*d_1_*): δ_H_ [p.p.m] (*J* [Hz]) 0.64 (3 H, d, *J* 6.9 Hz, H-30), 0.78 (3 H, d, *J* 6.4, H-32), 0.82 ( 3 H, d, *J* 6.3, H-28), 0.85 (3 H, t, *J* 7.0, H-25), 0.91 (3H, d, *J* 6.9, H-26), 1.03 (3 H, d, *J* 7.3, H-31), 1.05 (3 H, d, *J* 7.5, H-33), 1.07 (3 H, d, *J* 7.5, H-29), 1.21 (2 H, m, H-23), 1.22 (1 H, m, H-5), 1.27 (1 H, m, H-15), 1.27 (2 H, m, H-24), 1.32 (1 H, m, H-14), 1.48 (1 H, m, H-5), 1.50 (1 H, m, H-6), 1.57 (3 H, s, H-27), 1.64 (2 H, m, H-4), 1.66 (1 H, m, H-12), 1.74 (1 H, m, H-14), 1.98 (1 H, m, H-10), 2.00 (1 H, m, H-8), 2.17 (1 H, m, H-15), 2.21 (1 H, m, H-18), 2.41 (1 H, m, H-22), 3.20 (1 H, m, H-2), 3.41 (1 H, m, H-9), 3.53 (1 H, d, *J* 9.2, H-19), 3.60 (1 H, dd, *J* 8.9, 1.9, H-11), 3.70 (3 H, s, H-34), 3.73 (1 H, d, *J* 10.1, H-7), 4.06 (1 H, m, H-3), 4.07 (1 H, m, H-13), 5.08 (1 H, d, *J* 9.4, H-21), 5.32 (1 H, dd, *J* 15.3, 8.9, H-17), 5.47 (1 H, dt, *J* 15.3, 6.8, H-16). ^13^C NMR (125 MHz, chloroform-*d1*): δ_C_ [p.p.m] (*J* [Hz]) 11.0 (C-27), 11.4 (C-29), 11.5 (C-31), 13.5 (C-30), 14.4 (C-25), 15.3 (C-33), 17.7 (C-28), 17.9 (C-32), 20.9 (C-24), 21.2 (C-26), 25.2 (C-4), 26.5 (C-5), 29.3 (C-15), 31.9 (C-6), 32.0 (C-22), 34.2 (C-8), 34.7 (C-14), 37.7 (C-12), 38.7 (C-10), 39.8 (C-2), 40.1 (C-23), 42.1 (C-18), 52.6 (C-34), 69.1 (C-13), 74.8 (C-3), 76.3 (C-7), 82.1 (C-19), 84.2 (C-11), 84.6 (C-9), 131.1 (C-20), 133.5 (C-16), 133.6 (C-17), 135.9 (C-21), 175.8 (C-1). HR-ESIMS: *m/z* 605.4384 [M+Na]^+^, calcd 605.4388 for C_34_H_62_O_7_Na.

### 2.6 Cytotoxicity assays

The cytotoxicity of zincophorin, methyl zincophorin, daptomycin (Chemos GmbH, Regenstauf, Germany), and imipenem (Sigma-Aldrich GmbH, Taufkirchen, Germany) was evaluated as described recently [19], [20]. Briefly, serial twofold compounds concentration were prepared in Eagle’s minimum essential medium supplemented with 2 mM L-glutamine or Dulbecco’s Modified Eagle Medium (Lonza Group Ltd., Basel, Switzerland) and added to three-day-old confluent human lung carcinoma cells (A549; Institute of Molecular Virology, University of Münster, Germany) and Madin-Darby canine kidney cell (MDCK; Friedrich-Loeffler Institute, Riems, Germany) monolayers grown in 96-well plates (Greiner bio-one GmbH, Frickenhausen, Germany), respectively. Maximum tested compound concentration was 100 µM. After 72 h of incubation at 37°C with 5% CO_2_, cells were fixed and stained with a crystal violet solution. After dye elution and optical density measurement the 50% cytotoxic concentration (CC_50_) was calculated.

### 2.7 Antimicrobial assays with Streptococcus pneumoniae

The antimicrobial activity of zincophorin and its methyl ester was determined against pneumococcal reference strains DSM20566 and DSM14378 (serotype 1, ATCC 33400 and serotype 5, ATCC 6305, respectively; Leibniz Institute DSMZ – German Collection of Microorganisms and Cell Cultures, Braunschweig, Germany) as well as D39 (serotype 2, kindly provided by H. Slevogt, ZIK Septomics, Jena, Germany). Furthermore, four clinical isolates (6937, 9400, 8919, 8828) collected from patients with different symptoms in the Department of Medicinal Microbiology, Jena University Hospital [20], were included in these studies. Daptomycin and imipenem were used as control antibiotics able to inhibit both bacterial growth and biofilm formation [21], [22], [23], [24]. 

Bacteria were cultivated on Columbia blood agar plates with 5% sheep blood at 37°C in an atmosphere enriched with 5% CO_2_ overnight and grown to mid-exponential growth phase in brain heart infusion broth (BHI). For determination of bacterial growth (minimal inhibitory concentration: MIC) and biofilm inhibition (minimal biofilm inhibitory concentration: MBIC) samples of precultured pneumococci were diluted in BHI to a McFarland of 0.5 (1.5×10^8^ cfu/ml). Antimicrobial assays were conducted as published previously [20], [21], [22], [23], [24], [25]. Briefly, for microtiter broth microdilution assay 96-well V-shape plates (Greiner bio-one GmbH) with pneumococci in BHI and serial compound dilutions (dilution factor 2; maximum tested concentration of 50 µM) were incubated overnight at 37°C with 5% CO_2_ for 18 h. The planktonic growth of pneumococci was evaluated by measuring optical density at 620 nm. MIC was defined as the lowest compound concentration that reduced the turbidity by ≥90%. Biofilm inhibition assay was performed in 96-well F-bottom plates (Greiner bio-one GmbH) with pneumococci diluted in tryptic soy broth (TSB) for 2 h at 37°C with 5% CO_2_. Then, supernatant was replaced by TSB with diluted compound (maximal 50 µM). After further 24 h of incubation, crystal violet staining, dye elution, and optical density measurement were performed to quantify biofilm growth [20]. MBIC was defined as the lowest drug concentration that inhibited biofilm formation ≥90% compared to the mean value of 6 untreated controls (set as 100% growth). 

## 3 Results

### 3.1 Identification of the zincophorin biosynthesis gene cluster

The majority of bacterial polyketide synthases (PKS) are large, modularly organized biosynthetic enzymes, which inhere a template-based assembly strategy [26], [27]. Starting from an N-terminal PKS module, an acyl precursor is successively extended by decarboxylative Claisen condensations through a series of elongation modules. During this process, the growing acyl chain is covalently bound to the PKS by a thioester bond. Eventually, a C-terminal termination module releases the mature polyketide from its thioester linkage [27]. PKS are typically composed of a variable set of catalytic domains. Their activities range from the selection of acyl-coenzyme A (CoA) units to the processing of the Claisen-derived β-keto groups. While reductive domains in a module indicate different degrees of β-keto processing, the acyl transferase (AT) domains provide information on the respective substrates being incorporated. The close correlation between the PKS architecture, on the one hand, and the structure of the associated natural product, on the other, became known as the principle of colinearity [28]. In case of strict colinearity, the chemical constitution of a polyketide can be deduced from an analysis of its biosynthetic enzymes [29], [30]. Conversely, the structure of an isolated natural product can also be used to predict the catalytic domains, which constitute the corresponding molecular assembly line [16], [31].

The structure of zincophorin can be formally dissected into 12 biosynthetic building blocks, including a propionate starter unit as well as three C_2_- and eight C_3_-extender units. The latter would derive from malonyl-CoA and methylmalonyl-CoA, respectively. Since each module is responsible for the incorporation of a single building block in non-iterative PKS, the draft genome sequence of *S. griseus* HKI 0741 was screened for gene loci featuring 12 PKS modules [32]. Only one of the identified gene clusters satisfied this criterion and was therefore analyzed in detail. The substrate specificity of the gate-keeping AT domains [33], [34] and the number and type of reductive domains were already in perfect agreement with the structure of zincophorin (Table 1 (Tab. 1)). Yet, the presence of a β-ketoacyl synthase (KS) domain in the assumed loading module hampered an unequivocal assignment. Sequence alignments then revealed that the corresponding KS domain lacks an essential cysteine in its active site, but harbored a glutamine instead. According to literature data, this substitution has functional implications [35]. While prototypical KS domains perform chain elongation reactions, a glutamine-featuring KS_Q_ domain has decarboxylase activity towards acyl carrier protein (ACP)-bound substrates. Furthermore, acyl-ACP decarboxylation was shown to represent an alternative mechanism for the initiation of polyketide biosynthesis [35]. The identified zincophorin assembly line is hence a textbook example of colinear polyketide biosynthesis.

**Figure 2:**
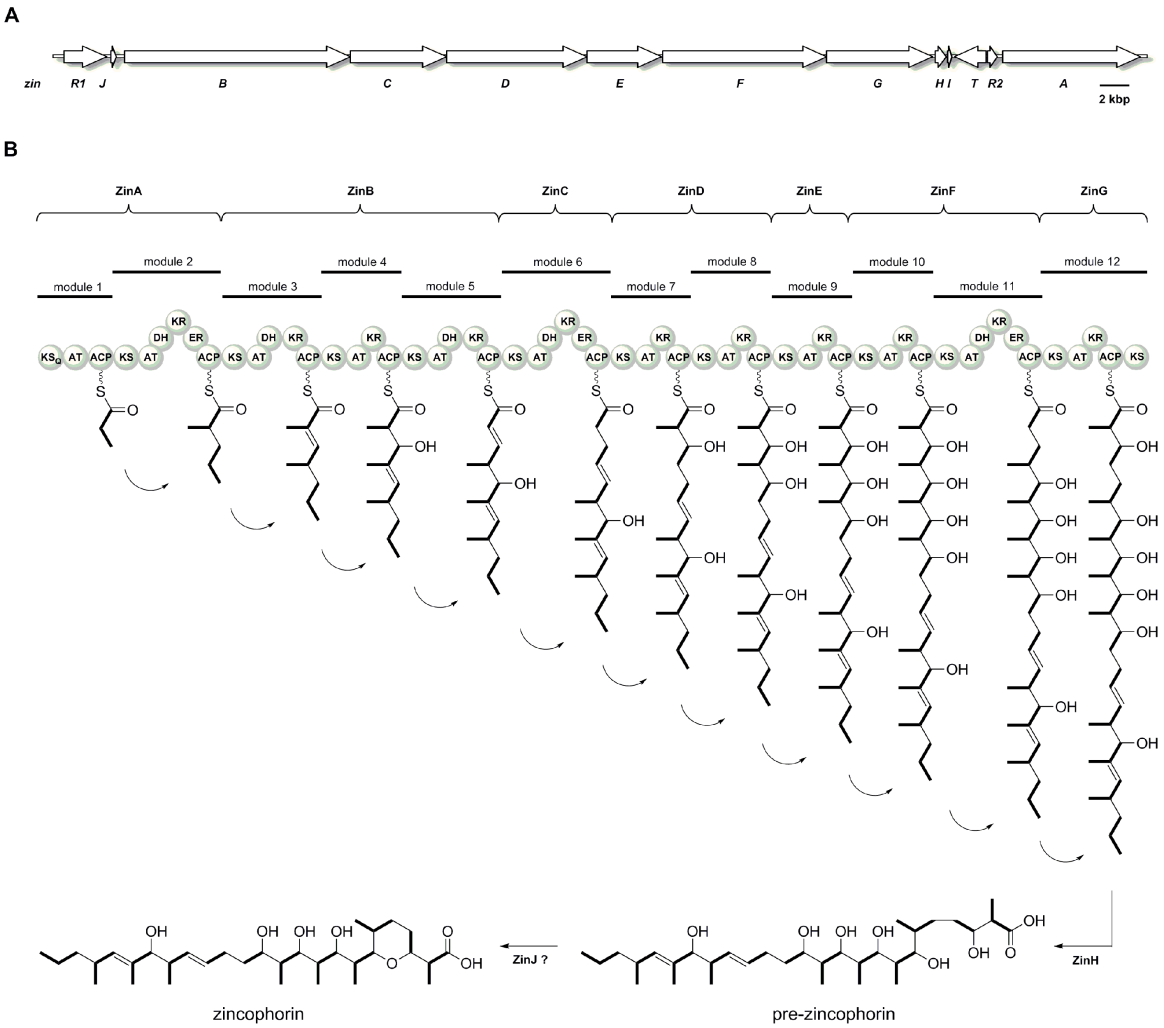
(A) Organization of the zincophorin biosynthesis gene cluster. (B) Molecular assembly line deduced from *zinA* to *zinG* and proposed biosynthesis of zincophorin via the intermediate pre-zincophorin. The acyl-CoA-derived C_2_ and C_3_ building blocks are highlighted in bold. The domain notation is given in Table 1.

The zincophorin gene cluster covers 73.5 kbp of contiguous DNA on the chromosome of strain HKI 0741 (Figure 2 (Fig. 2)). All attempts to inactivate the zincophorin gene cluster in *S. griseus* HKI 0741 by means of allelic replacement remained unsuccessful. It harbors 13 discrete ORFs, including seven PKS genes (*zinA–zinG*), a transport gene (*zinT*), and two regulatory genes (*zinR1*, *zinR2*). The ORF *zinH* was annotated as a hydrolase. Because the terminating PKS module of ZinG lacks a thioesterase domain, ZinH is expected to mediate the hydrolytic offload of the fully assembled acyl chain. Homology alignments of *zinJ* indicate a possible relationship to epoxide hydrolase genes, which are involved in polyether ring formations [36], [37], whereas *zinI* was predicted to encode a histidine kinase. A subsequent analysis of bacterial genomes that were deposited in GenBank revealed that the zincophorin biosynthesis gene cluster is fully conserved in *S. griseus* IFO 13350 [38], [39], where it spans the genes SGR6071-SGR6083. Strain IFO 13350 is well-known for the production of streptomycin. According to the present study, it might also harbor the genetic potential for the biosynthesis of zincophorin.

### 3.2 Antipneumococcal activity and cytotoxic effects of zincophorin and its methyl ester

For biological testing, zincophorin was isolated from fermentation cultures of *S. griseus* HKI 0741. For the methylation of the purified natural product the toxic and explosive reagent diazomethane [8] was replaced by trimethylsilyldiazomethane. Its methyl ester was synthesized following a described methylation protocol [40].

In the antimicrobial assays, zincophorin was very effective in inhibiting growth and biofilm formation of all tested *S. pneumoniae* strains (Table 2 (Tab. 2)). 

**Figure 3:**
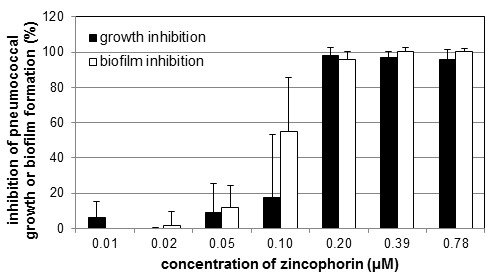
Dose dependence of the effect of zincophorin on growth and biofilm formation of *S. pneumoniae* DSM20566. Mean values of the inhibitory effect in % with standard deviation of at least 3 tests each with 2 parallels are shown.

MIC values ranged from 0.09 to 0.21 µM and were thus in the same order of magnitude as those of daptomycin, albeit higher than those of imipenem. The only exception was observed for the clinical *S. pneumoniae* isolate 8919, which was more susceptible to zincophorin and daptomycin. The antibacterial effects of zincophorin were dose-dependent, as exemplarily shown for reference strain DSM20566 in Figure 3 (Fig. 3). In terms of biofilm inhibition, zincophorin was clearly superior to daptomycin. Interestingly, the methyl ester of zincophorin exhibited no antibacterial effect up to 50 µM (data not shown), indicating that the carboxylic acid function of zincophorin is crucial for the activity against pneumococci. Subsequently, we explored the impact of all test compounds on the viability of A549 and MDCK cells. 

While the reference antibiotics and zincophorin methyl ester were tolerated up to 100 µM, zincophorin exerted strong cytotoxic effects in both cell lines (Table 3 (Tab. 3)). 

## 4 Discussion

**Figure 4:**
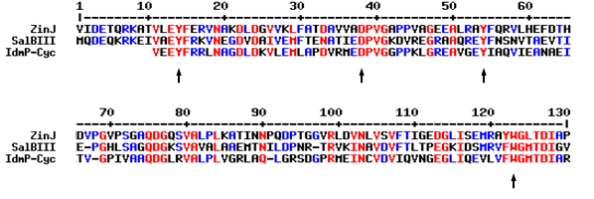
MultAlin [51] sequence alignment of ZinJ with the epoxide hydrolases SalBIII and IdmP-Cyc from salinomycin and indanomycin biosynthesis [45], [46]. Selected amino acid residues of the active site cavity [44] are indicated with arrows.

The gene cluster for the biosynthesis of zincophorin was discovered by genome mining, exploiting the predictability of modular polyketide assembly [16], [28], [29], [30]. The intermediate pre-zincophorin, which is offloaded from the proposed assembly line, features the necessary carbon backbone, and it also possesses the substitution and oxidative pattern to be expected (Figure 2 (Fig. 2)). In contrast, the intramolecular cyclization to the tetrahydropyran ring in zincophorin is enigmatic. Nature provides different strategies for the formation of oxygen-containing heterocycles [41], [42]. In the biosynthesis of polyether ionophores, alkene functionalities of a linear polyketide are converted into epoxides, before a series of successive S_N_2 ring openings give rise to the polycyclic structure of the final product [43]. Although the presence of a putative epoxide hydrolase gene in the *zin* locus initially suggested a related mechanism, we did not detect any epoxidase gene in the entire genome of *S. griseus* HKI 0741 to strengthen this hypothesis. The absence of a dehydratase (DH) domain in modules 10 and 12 further opposes this scenario, albeit a non-colinear action of a remote DH domain cannot be completely ruled out. Likewise, the lack of a DH domain in module 12 would be inconsistent with a Michael addition to produce the O heterocycle, as a Δ^2,3^ double bond in pre-zincophorin would be a prerequisite for this reaction. Another common mechanism for the formation of an ether ring involves the nucleophilic addition of an alcohol to a carbonyl group, yielding a cyclic hemiketal. However, all KR domains of the zincophorin assembly line possess an intact NADPH-binding pocket and appear to be fully functional [44]. Therefore, the presence of a carbonyl group at C-3 or C-7 in pre-zincophorin appears rather unlikely. A literature search revealed that the same discrepancy between PKS domain architecture and the occurrence of a tetrahydropyran ring was previously observed in the biosyntheses of indanomycin and salinomycin [45], [46]. Interestingly, both pathways were also found to feature a single ZinJ-like epoxide hydrolase. In case of indanomycin, the corresponding enzyme has been implicated in the oxa-conjugate addition [45]. Recently, biochemical analysis as well as structure-guided mutagenesis confirmed pyran synthase activity of the ZinJ homolog from salinomycin biosynthesis [47]. Key residues which constitute the active site cavity of the latter are conserved in ZinJ (Figure 4 (Fig. 4)), suggesting a similar catalytic mechanism.

In this study, we demonstrated that the intrinsic antimicrobial activity of zincophorin extends to *Streptococcus pneumoniae*, which is the leading cause of bacterial pneumonia [48]. In general, MIC and MBIC values of zincophorin were higher as compared to the reference imipenem, but slightly superior to those of daptomycin. All tested *S. pneumoniae* strains were equally susceptible to zincophorin, whereas the activity of imipenem was significantly reduced against strain 8919. The latter had been isolated from a cystic fibrosis patient and is resistant towards β-lactam antibiotics. Antibacterial activity of zincophorin was completely abolished upon esterification of its carboxylate group, which is consistent with the previously proposed ionophore function of the polyketide [7]. Ionophore antibiotics are known to possess broad cytotoxic effects [49]. Noteworthy, chemical modification of the ionophore salinomycin was recently reported to alleviate toxicity while preserving the antibacterial activity [50]. The identification of the *zin* gene cluster could hence set the stage for producing zincophorin derivatives with an improved toxicity profile using genetic engineering instead of chemical synthesis. 

## Notes

### Authorship

EW, SB and HK contributed equally to this study.

### Competing interests

The authors declare that they have no competing interests.

### Funding

Financial support was provided by the Bundesministerium für Bildung und Forschung (BMBF) within the programme InfectControl 2020, the European Social Fund (ESF) and the Thuringian Ministry of Economy, Labor and Technology (TMWAT; 2011FGR0137).

### Acknowledgements

We thank Andrea Perner and Heike Heinecke for recording HR-ESI-MS and NMR spectra.

## Figures and Tables

**Table 1:**
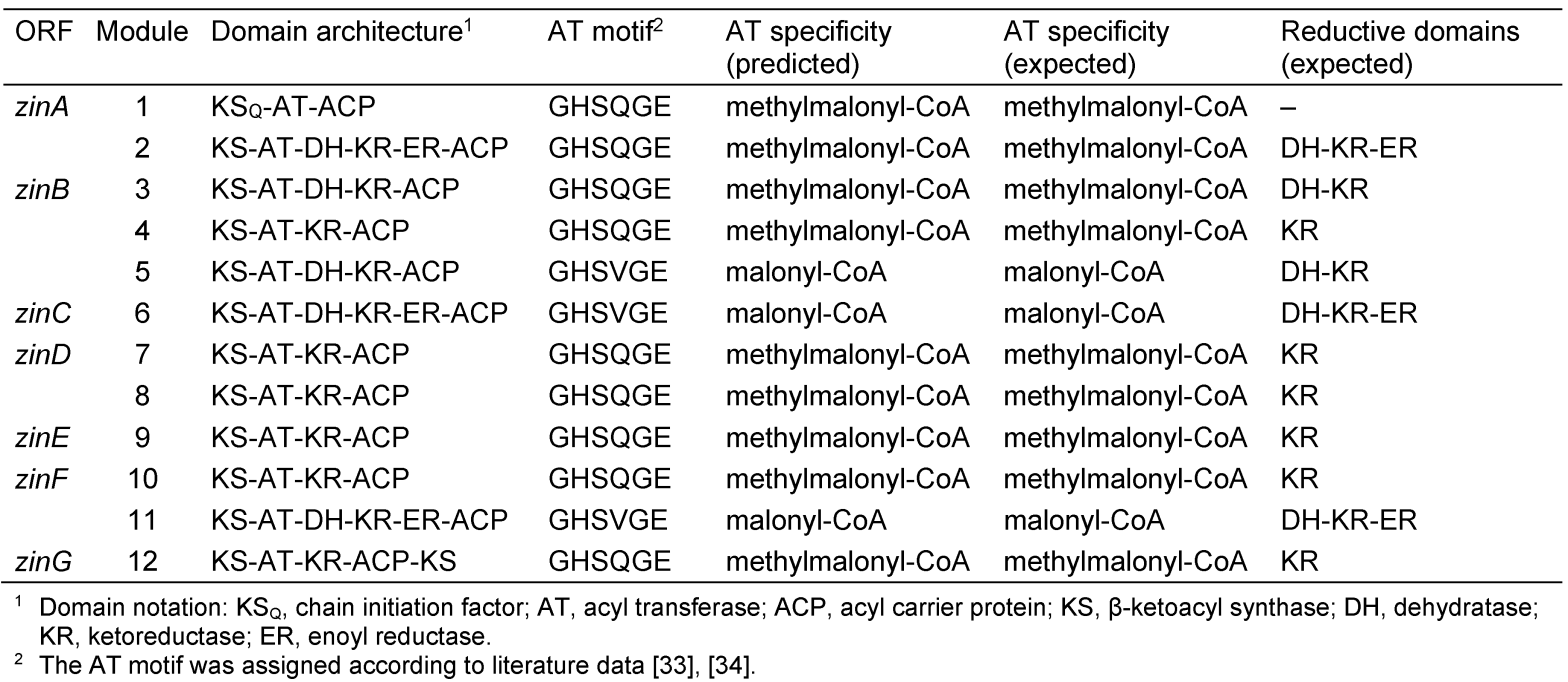
PKS assembly line for the biosynthesis of zincophorin

**Table 2:**
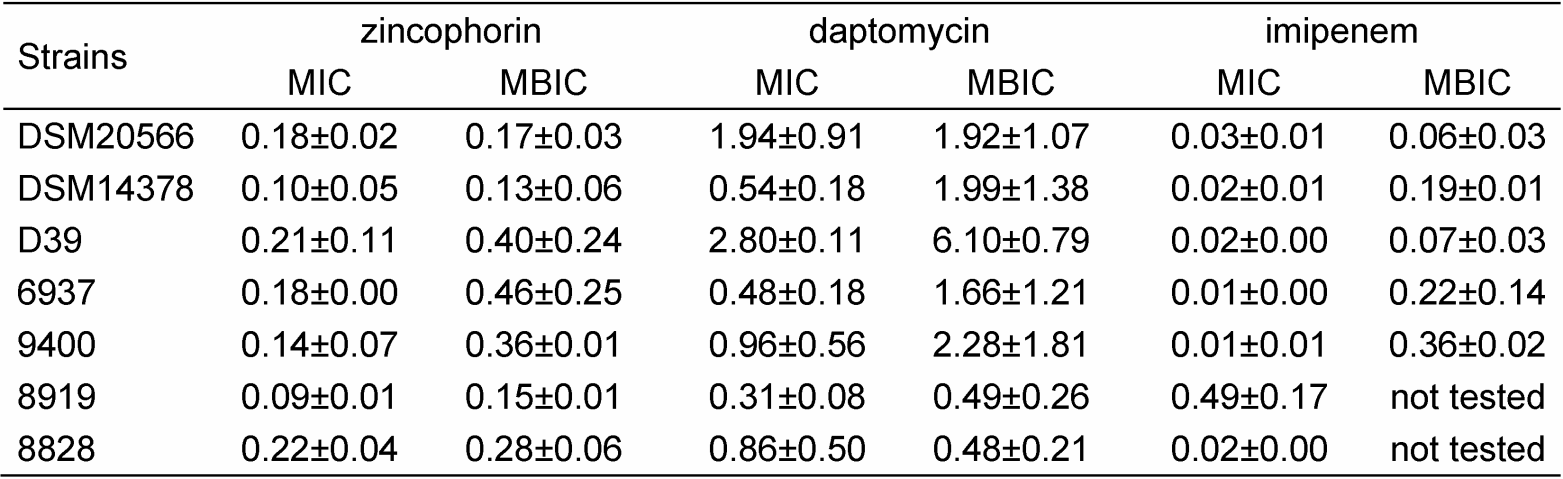
Antibacterial activity of zincophorin, daptomycin, and imipenem. Mean MIC and MBIC values in μM with standard deviation determined for three reference strains and four clinical isolates of *S. pneumoniae* in microtiter broth dilution and biofilm assay, respectively, are shown.

**Table 3:**
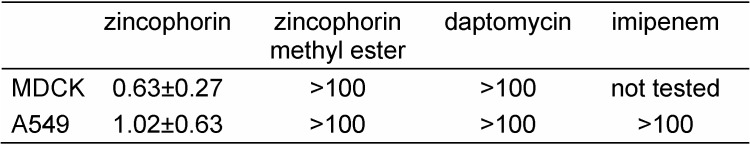
Cytotoxicity of zincophorin, zincophorin methyl ester, daptomycin, and imipenem. Mean values of the 50% cytotoxic concentrations in µM (CC_50_) with standard deviation are shown.

## References

[1] Genilloud O (2014). The re-emerging role of microbial natural products in antibiotic discovery. Antonie Van Leeuwenhoek.

[2] Müller R, Wink J (2014). Future potential for anti-infectives from bacteria – how to exploit biodiversity and genomic potential. Int J Med Microbiol.

[3] Pidot SJ, Coyne S, Kloss F, Hertweck C (2014). Antibiotics from neglected bacterial sources. Int J Med Microbiol.

[4] Cragg GM, Grothaus PG, Newman DJ (2014). New horizons for old drugs and drug leads. J Nat Prod.

[5] Butler MS, Blaskovich MA, Cooper MA (2013). Antibiotics in the clinical pipeline in 2013. J Antibiot.

[6] Eisenstein BI, Oleson FB, Baltz RH (2010). Daptomycin: from the mountain to the clinic, with essential help from Francis Tally, MD. Clin Infect Dis.

[7] Brooks HA, Gardner D, Poyser JP, King TJ (1984). The structure and absolute stereochemistry of zincophorin (antibiotic M144255): a monobasic carboxylic acid ionophore having a remarkable specificity for divalent cations. J Antibiot.

[8] Gräfe U, Schade W, Roth M, Radics L, Incze M, Ujszászy K (1984). Griseochelin, a novel carboxylic acid antibiotic from Streptomyces griseus. J Antibiot.

[9] Song Z, Lohse AG, Hsung RP (2009). Challenges in the synthesis of a unique mono-carboxylic acid antibiotic, (+)-zincophorin. Nat Prod Rep.

[10] Harrison TJ, Ho S, Leighton JL (2011). Toward more “ideal” polyketide natural product synthesis: a step-economical synthesis of zincophorin methyl ester. J Am Chem Soc.

[11] Wu MC, Law B, Wilkinson B, Micklefield J (2012). Bioengineering natural product biosynthetic pathways for therapeutic applications. Curr Opin Biotechnol.

[12] Kreutzer MF, Kage H, Herrmann J, Pauly J, Hermenau R, Müller R, Hoffmeister D, Nett M (2014). Precursor-directed biosynthesis of micacocidin derivatives with activity against Mycoplasma pneumoniae. Org Biomol Chem.

[13] Ueberschaar N, Xu Z, Scherlach K, Metsä-Ketelä M, Bretschneider T, Dahse HM, Görls H, Hertweck C (2013). Synthetic remodeling of the chartreusin pathway to tune antiproliferative and antibacterial activities. J Am Chem Soc.

[14] Chin CS, Alexander DH, Marks P, Klammer AA, Drake J, Heiner C, Clum A, Copeland A, Huddleston J, Eichler EE, Turner SW, Korlach J (2013). Nonhybrid, finished microbial genome assemblies from long-read SMRT sequencing data. Nat Methods.

[15] Eid J, Fehr A, Gray J, Luong K, Lyle J, Otto G, Peluso P, Rank D, Baybayan P, Bettman B, Bibillo A, Bjornson K, Chaudhuri B, Christians F, Cicero R, Clark S, Dalal R, Dewinter A, Dixon J, Foquet M, Gaertner A, Hardenbol P, Heiner C, Hester K, Holden D, Kearns G, Kong X, Kuse R, Lacroix Y, Lin S, Lundquist P, Ma C, Marks P, Maxham M, Murphy D, Park I, Pham T, Phillips M, Roy J, Sebra R, Shen G, Sorenson J, Tomaney A, Travers K, Trulson M, Vieceli J, Wegener J, Wu D, Yang A, Zaccarin D, Zhao P, Zhong F, Korlach J, Turner S (2009). Real-time DNA sequencing from single polymerase molecules. Science.

[16] Nett M (2014). Genome mining: concept and strategies for natural product discovery. Prog Chem Org Nat Prod.

[17] Ishikawa J, Hotta K (1999). FramePlot: a new implementation of the frame analysis for predicting protein-coding regions in bacterial DNA with a high G + C content. FEMS Microbiol Lett.

[18] Udwary DW, Zeigler L, Asolkar RN, Singan V, Lapidus A, Fenical W, Jensen PR, Moore BS (2007). Genome sequencing reveals complex secondary metabolome in the marine actinomycete Salinispora tropica. Proc Natl Acad Sci USA.

[19] Schmidtke M, Schnittler U, Jahn B, Dahse H, Stelzner A (2001). A rapid assay for evaluation of antiviral activity against coxsackie virus B3, influenza virus A, and herpes simplex virus type 1. J Virol Methods.

[20] Walther E, Richter M, Xu Z, Kramer C, von Grafenstein S, Kirchmair J, Grienke U, Rollinger JM, Liedl KR, Slevogt H, Sauerbrei A, Saluz HP, Pfister W, Schmidtke M (2015). Antipneumococcal activity of neuraminidase inhibiting artocarpin. Int J Med Microbiol.

[21] Fitoussi F, Doit C, Benali K, Bonacorsi S, Geslin P, Bingen E (1998). Comparative in vitro killing activities of meropenem, imipenem, ceftriaxone, and ceftriaxone plus vancomycin at clinically achievable cerebrospinal fluid concentrations against penicillin-resistant Streptococcus pneumoniae isolates from children with meningitis. Antimicrob Agents Chemother.

[22] Pankuch GA, Jacobs MR, Appelbaum PC (2003). Postantibiotic effects of daptomycin against 14 staphylococcal and pneumococcal clinical isolates. Antimicrob Agents Chemother.

[23] Pankuch GA, Jacobs MR, Appelbaum PC (2003). Bactericidal activity of daptomycin against Streptococcus pneumoniae compared with eight other antimicrobials. J Antimicrob Chemother.

[24] Rybak MJ (2006). The efficacy and safety of daptomycin: first in a new class of antibiotics for Gram-positive bacteria. Clin Microbiol Infect.

[25] Grienke U, Richter M, Walther E, Hoffmann A, Kirchmair J, Makarov V, Nietzsche S, Schmidtke M, Rollinger JM (2016). Discovery of prenylated flavonoids with dual activity against influenza virus and Streptococcus pneumoniae. Sci Rep.

[26] Gulder TA, Freeman MF, Piel J (2011). The Catalytic Diversity of Multimodular Polyketide Synthases: Natural Product Biosynthesis Beyond Textbook Assembly Rules. Top Curr Chem.

[27] Hertweck C (2009). The biosynthetic logic of polyketide diversity. Angew Chem Int Ed Engl.

[28] Donadio S, Staver MJ, McAlpine JB, Swanson SJ, Katz L (1991). Modular organization of genes required for complex polyketide biosynthesis. Science.

[29] Banskota AH, Mcalpine JB, Sørensen D, Aouidate M, Piraee M, Alarco AM, Omura S, Shiomi K, Farnet CM, Zazopoulos E (2006). Isolation and identification of three new 5-alkenyl-3,3(2H)-furanones from two streptomyces species using a genomic screening approach. J Antibiot.

[30] McAlpine JB, Bachmann BO, Piraee M, Tremblay S, Alarco AM, Zazopoulos E, Farnet CM (2005). Microbial genomics as a guide to drug discovery and structural elucidation: ECO-02301, a novel antifungal agent, as an example. J Nat Prod.

[31] Schieferdecker S, König S, Weigel C, Dahse HM, Werz O, Nett M (2014). Structure and biosynthetic assembly of gulmirecins, macrolide antibiotics from the predatory bacterium Pyxidicoccus fallax. Chemistry.

[32] Weber T, Blin K, Duddela S, Krug D, Kim HU, Bruccoleri R, Lee SY, Fischbach MA, Müller R, Wohlleben W, Breitling R, Takano E, Medema MH (2015). antiSMASH 3.0-a comprehensive resource for the genome mining of biosynthetic gene clusters. Nucleic Acids Res.

[33] Haydock SF, Aparicio JF, Molnár I, Schwecke T, Khaw LE, König A, Marsden AF, Galloway IS, Staunton J, Leadlay PF (1995). Divergent sequence motifs correlated with the substrate specificity of (methyl)malonyl-CoA:acyl carrier protein transacylase domains in modular polyketide synthases. FEBS Lett.

[34] Reeves CD, Murli S, Ashley GW, Piagentini M, Hutchinson CR, McDaniel R (2001). Alteration of the substrate specificity of a modular polyketide synthase acyltransferase domain through site-specific mutations. Biochemistry.

[35] Bisang C, Long PF, Cortés J, Westcott J, Crosby J, Matharu AL, Cox RJ, Simpson TJ, Staunton J, Leadlay PF (1999). A chain initiation factor common to both modular and aromatic polyketide synthases. Nature.

[36] Harvey BM, Mironenko T, Sun Y, Hong H, Deng Z, Leadlay PF, Weissman KJ, Haydock SF (2007). Insights into polyether biosynthesis from analysis of the nigericin biosynthetic gene cluster in Streptomyces sp. DSM4137. Chem Biol.

[37] Shichijo Y, Migita A, Oguri H, Watanabe M, Tokiwano T, Watanabe K, Oikawa H (2008). Epoxide hydrolase Lsd19 for polyether formation in the biosynthesis of lasalocid A: direct experimental evidence on polyene-polyepoxide hypothesis in polyether biosynthesis. J Am Chem Soc.

[38] Nett M, Ikeda H, Moore BS (2009). Genomic basis for natural product biosynthetic diversity in the actinomycetes. Nat Prod Rep.

[39] Ohnishi Y, Ishikawa J, Hara H, Suzuki H, Ikenoya M, Ikeda H, Yamashita A, Hattori M, Horinouchi S (2008). Genome sequence of the streptomycin-producing microorganism Streptomyces griseus IFO 13350. J Bacteriol.

[40] Presser A, Hufner A (2004). Trimethylsilyldiazomethane – A mild and efficient reagent for the methylation of carboxylic acids and alcohols in natural products. Monatsh Chem.

[41] Richter ME, Traitcheva N, Knüpfer U, Hertweck C (2008). Sequential asymmetric polyketide heterocyclization catalyzed by a single cytochrome P450 monooxygenase (AurH). Angew Chem Int Ed Engl.

[42] Sundaram S, Hertweck C (2016). On-line enzymatic tailoring of polyketides and peptides in thiotemplate systems. Curr Opin Chem Biol.

[43] Gallimore AR (2009). The biosynthesis of polyketide-derived polycyclic ethers. Nat Prod Rep.

[44] Keatinge-Clay AT, Stroud RM (2006). The structure of a ketoreductase determines the organization of the beta-carbon processing enzymes of modular polyketide synthases. Structure.

[45] Li C, Roege KE, Kelly WL (2009). Analysis of the indanomycin biosynthetic gene cluster from Streptomyces antibioticus NRRL 8167. Chembiochem.

[46] Yurkovich ME, Tyrakis PA, Hong H, Sun Y, Samborskyy M, Kamiya K, Leadlay PF (2012). A late-stage intermediate in salinomycin biosynthesis is revealed by specific mutation in the biosynthetic gene cluster. Chembiochem.

[47] Luhavaya H, Dias MV, Williams SR, Hong H, de Oliveira LG, Leadlay PF (2015). Enzymology of Pyran Ring A Formation in Salinomycin Biosynthesis. Angew Chem Int Ed Engl.

[48] Krzysciak W, Pluskwa KK, Jurczak A, Koscielniak D (2013). The pathogenicity of the Streptococcus genus. Eur J Clin Microbiol Infect Dis.

[49] Rutkowski J, Brzezinski B (2013). Structures and properties of naturally occurring polyether antibiotics. Biomed Res Int.

[50] Antoszczak M, Popiel K, Stefanska J, Wietrzyk J, Maj E, Janczak J, Michalska G, Brzezinski B, Huczynski A (2014). Synthesis, cytotoxicity and antibacterial activity of new esters of polyether antibiotic – salinomycin. Eur J Med Chem.

[51] Corpet F (1988). Multiple sequence alignment with hierarchical clustering. Nucleic Acids Res.

